# Supplementary material for: Reproductive history differentially shapes the neural response of middle-aged hAPOEɛ4 female rats to estradiol therapy after a metabolic challenge
Source: Biol Sex Differ. 2026 May 3;17:123. doi: 10.1186/s13293-026-00911-y (PMC13285403; doi:10.1186/s13293-026-00911-y)

**Supplementary**


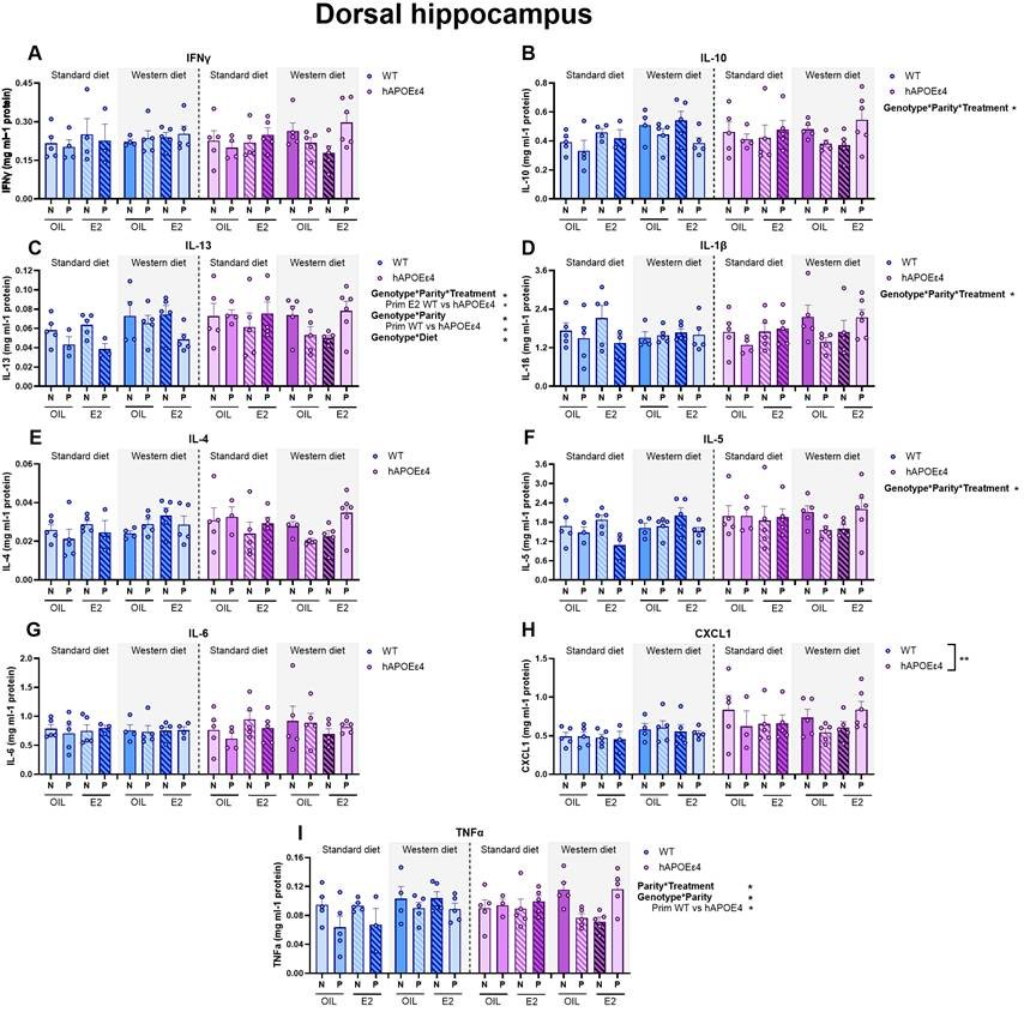


*Supplementary Figure 1.***Cytokines in the dorsal hippocampus.**Levels of interferon γ (IFNγ; A), interleukin (IL)-10 (B), IL-13 (C), IL-1β (D), IL-4 (E), IL-5 (F), IL-6 (G), chemokine (C-X-C motif) ligand 1 (CXCL1; H), tumor necrosis factor (TNF) α (I). n = 3-6 for treatment, genotype, parity and diet groups. ***p *< 0.05, ***p *<* 0.01. N = nulliparous; P = primiparous; WT = wildtype; E2 = estradiol.


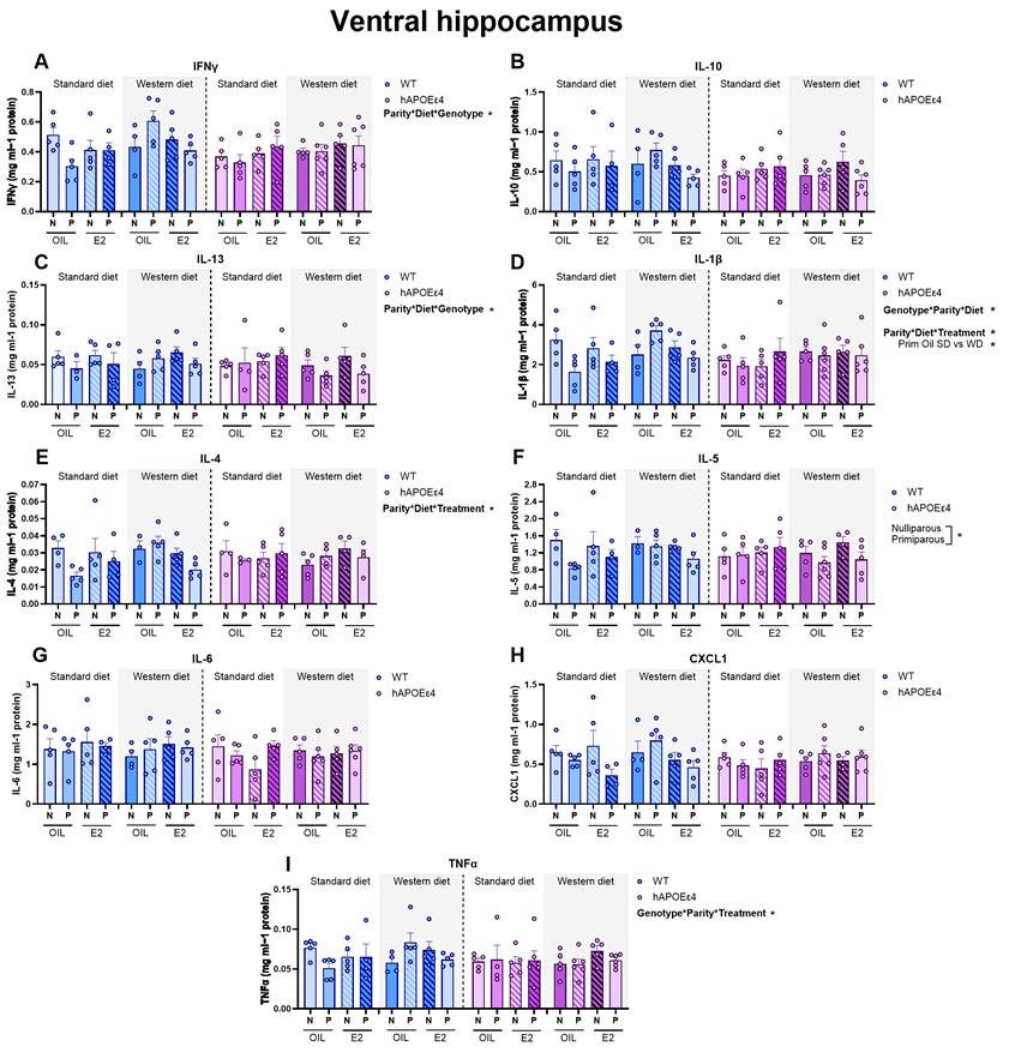


*Supplementary Figure 2.***Cytokines in the ventral hippocampus.**Levels of interferon γ (IFNγ; A), interleukin (IL)-10 (B), IL-13 (C), IL-1β (D), IL-4 (E), IL-5 (F), IL-6 (G), chemokine (C-X-C motif) ligand 1 (CXCL1; H), tumor necrosis factor (TNF) α (I). n = 3-6 for treatment, genotype, parity and diet groups. ***p *< 0.05*. N = nulliparous; P = primiparous; WT = wildtype; E2 = estradiol.


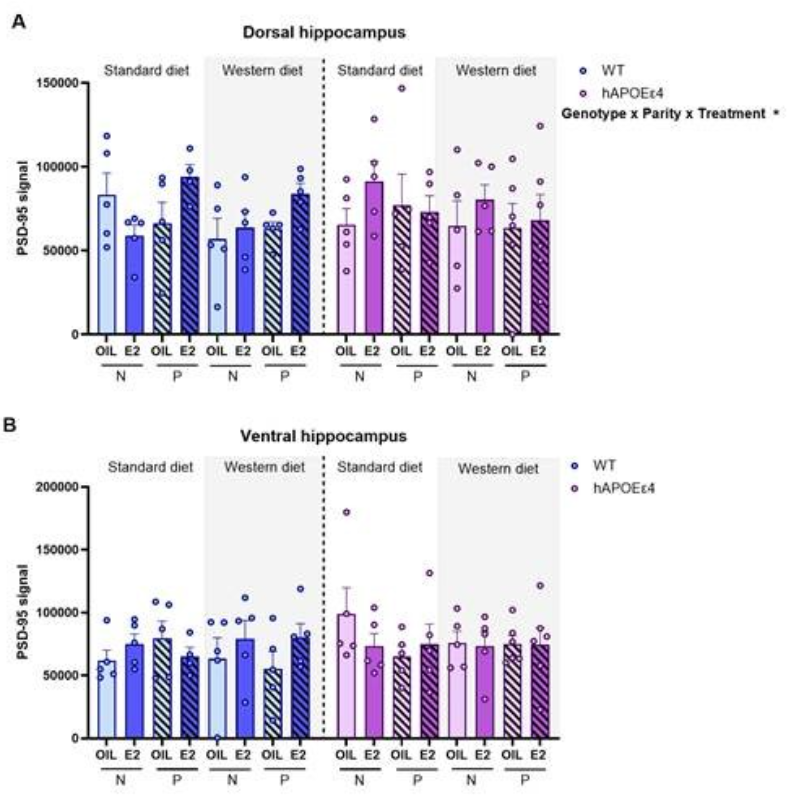


*Supplementary Figure 3.***Effects of genotype, parity, diet, and E2 treatment on synaptic plasticity.**PSD-95 signal in the dorsal (dHPC; A) and ventral hippocampus (vHPC: B) in middle-aged females relative to total protein. n = 4-6 for treatment, genotype, parity and diet groups. * p < 0.05, ** p < 0.005. E2 = estradiol, WT = wildtype, SD= standard diet, WD = western diet, N = nulliparous, P = primiparous.


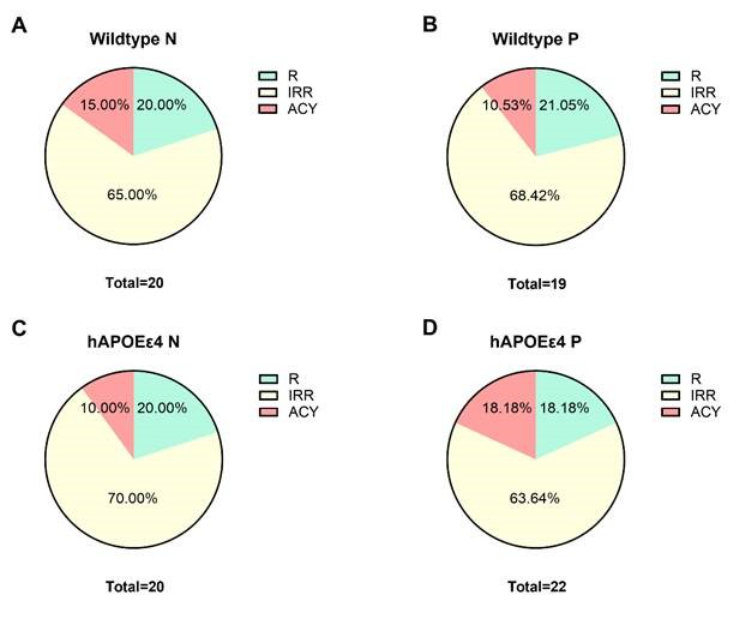


*Supplementary Figure 4*. **Estrous cycling before treatment (10-11 months of age).**Cycling in wildtype (WT) nulliparous (N; A), and primiparous (P; B), and in hAPOEɛ4 N (C) and hAPOEɛ4 P (D). R = regular cycling; IRR = Irregular cycling; ACY = Acyclic.


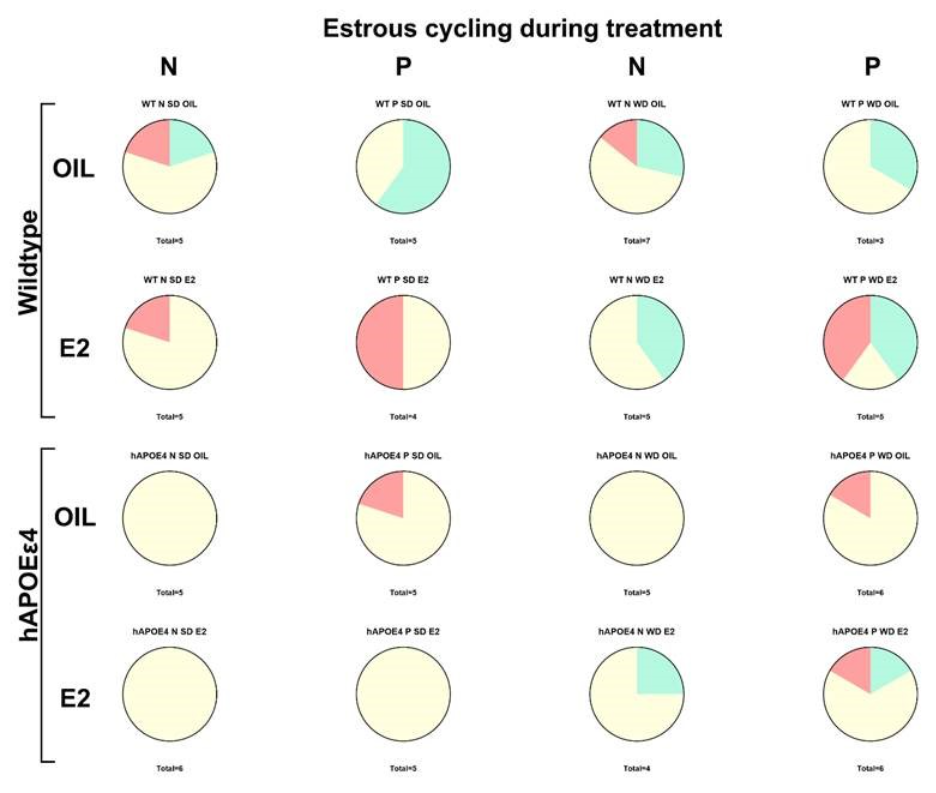


*Supplementary Figure 5.* **Estrous cycling during treatment (11-12 months of age).**Cycling in wildtype (WT) and hAPOEɛ4 females of nulliparous (N) or primiparous (P) parity, fed with Standard (SD) or western diet (WD) and treated with estradiol (E2) or vehicle (oil). R = regular cycling; IRR = Irregular cycling; ACY = Acyclic.


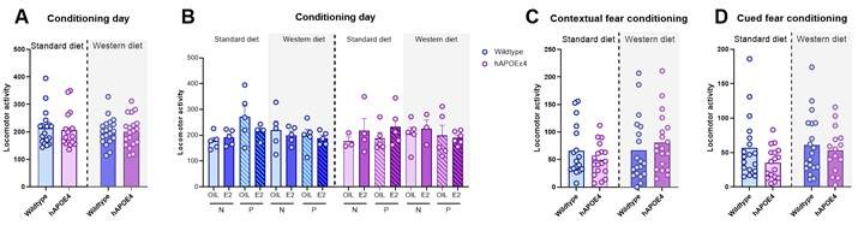


*Supplementary Figure 6.***No significant differences in locomotor activity were found during the fear conditioning task.** Locomotor activity during the conditioning day looking at genotype and diet effects (A), as well as with all groups broken down (B), contextual (C) and cued (D) fear conditioning tasks. n = 3-20. No genotype, parity, diet or treatment effects results in differences in locomotion (all p > 0.22) with the variation present being driven by freezing behavior.


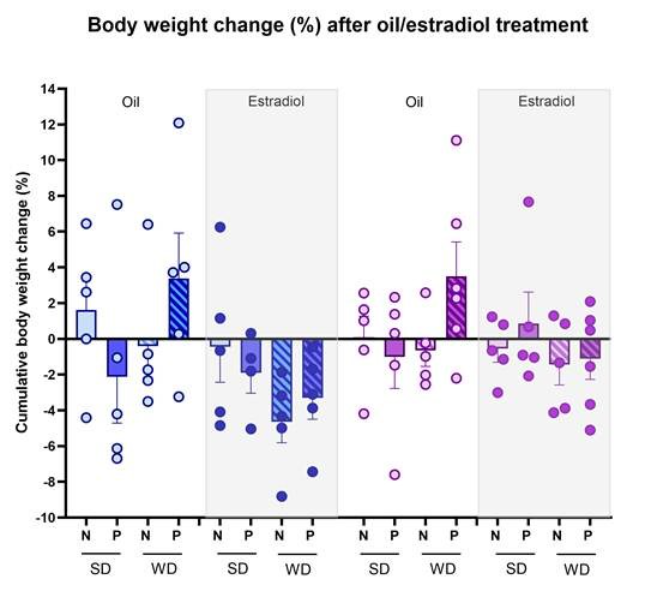


*Supplementary Figure 7.***Percent body weight change in % during oil/estradiol treatment period.** n = 9-10 females per genotype, parity and diet for diet only groups and 3-6 for treatment, genotype, parity and diet groups. N = nulliparous; P = primiparous; SD = standard diet; WD = Western diet.


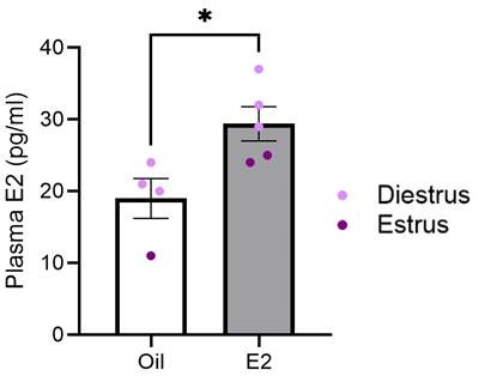


*Supplementary Figure 8.***Plasma estradiol (E2) levels in nulliparous WT groups that received either oil or E2.** n = 4-5 per group. * p < 0.05. Both groups included animals staged in diestrus and estrus, where E2 treatment produced consistently higher levels regardless of stage (oil diestrus average = 21.67; E2 diestrus average = 32.67). One oil injected animal was also in proestrus (28 pg/ml, irregular) ultimately capturing natural peaks in E2 levels.

*Table*1 Statistical details of cytokines loaded onto Principal Component (PC) 1 and 2 in the dorsal and ventral hippocampus.


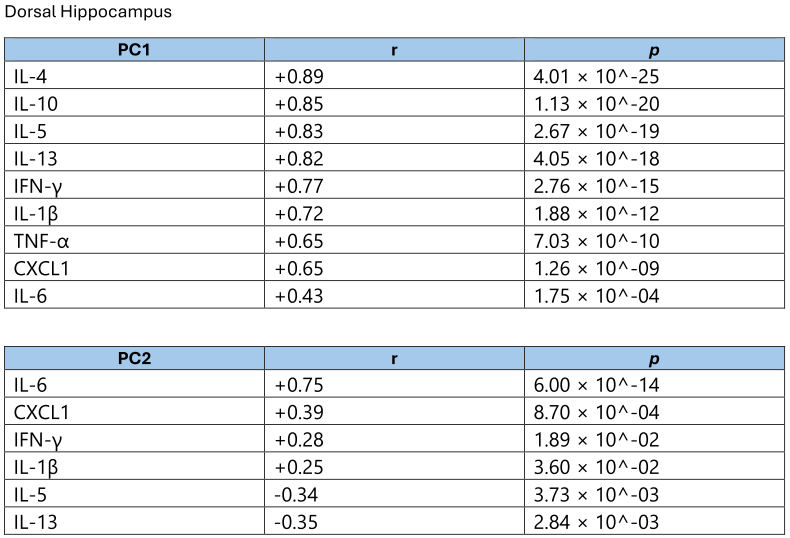


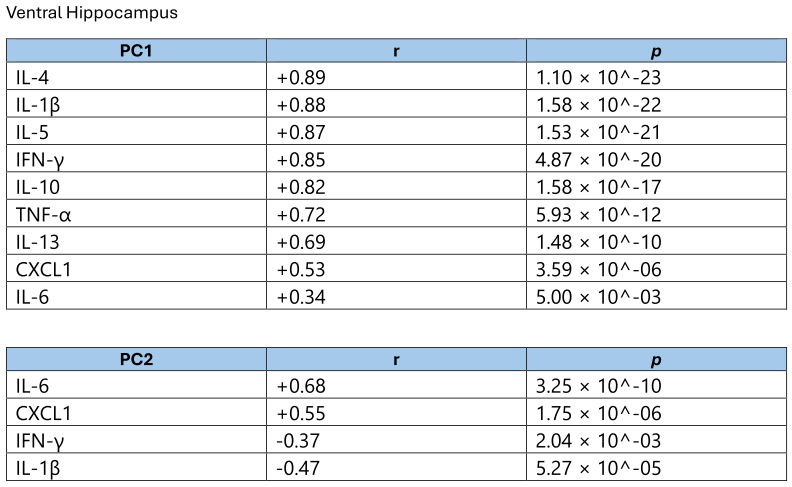


*Table*2 Statistical details of metabolic hormones loaded onto Principal Component (PC) 1 and 2 in plasma


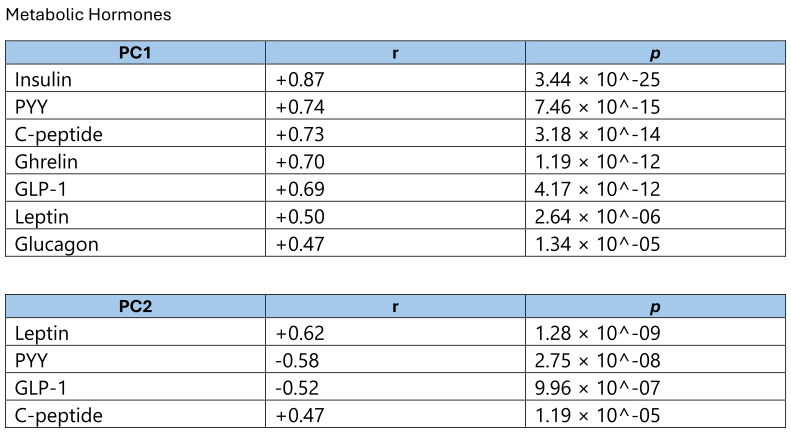


*Table 3*Individual loading percentages of each PC in the dorsal and ventral hippocampus

 
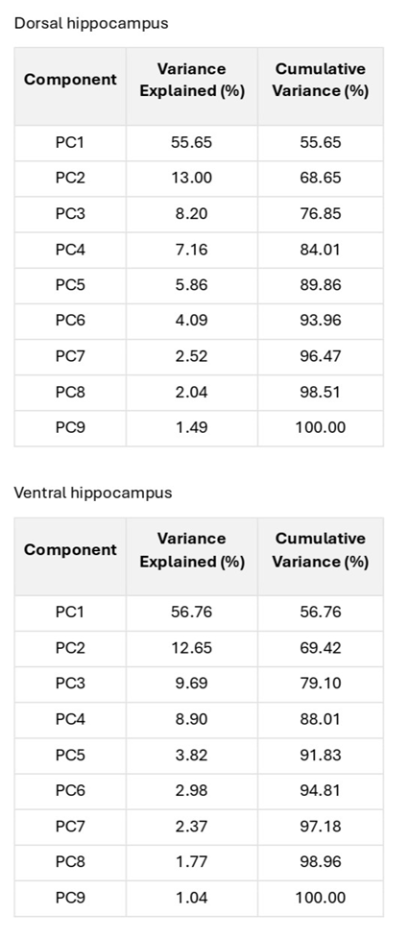


*Table 4*Individual cytokine levels in the dorsal and ventral hippocampus


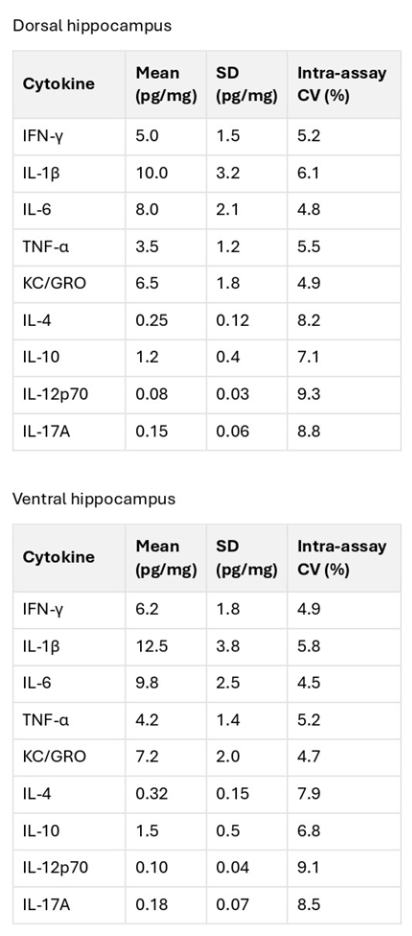

Supplement: Supplementary file 1 — Supplementary Material 1 [file 13293_2026_911_MOESM2_ESM.docx]
